# Psychological determinants associated with vaccination intentions acceptance during pandemic events

**DOI:** 10.1192/j.eurpsy.2022.1277

**Published:** 2022-09-01

**Authors:** A. Zartaloudi

**Affiliations:** University of West Attica, Nursing, Athens, Greece

**Keywords:** COVID-19 vaccination, vaccination intention, Psychological determinants, Vaccine acceptance

## Abstract

**Introduction:**

Psychological factors, like general self-efficacy, optimism or subjective well-being, might further enhance the understanding of why certain people vaccinate while others do not.

**Objectives:**

To identify psychological factors associated with people’s decision to vaccinate during pandemic events.

**Methods:**

A literature review has been made through PubMed database.

**Results:**

Psychology offers three general propositions for understanding and intervening to increase uptake where vaccines are available and affordable. The first proposition is that thoughts and feelings can motivate getting vaccinated. Low confidence in vaccine effectiveness and concern about safety correlate reliably with not getting vaccinated. The second proposition is that social processes can motivate getting vaccinated. Social norms are associated with vaccination. Recommendation by friends, mainstream media and social media affected vaccination intention. The third proposition is that interventions can facilitate vaccination directly by leveraging, but not trying to change, what people think and feel. To increase vaccine uptake, these interventions build on existing favorable intentions by facilitating action (through reminders, prompts, and primes) and reducing barriers (through logistics and healthy defaults); these interventions also shape behavior (through incentives, sanctions, and requirements). Perceived risk and effectiveness of the vaccine as well as trust in the government and health authorities was related to people’s vaccination intention.

**Conclusions:**

There are significant associations of general individual psychological constructs with the decision to vaccinate. This may provide useful frameworks for understanding the causal mechanisms behind this relationship, which could help to develop intervention strategies to effectively promote vaccination intentions that increase vaccination rates among population.

**Disclosure:**

No significant relationships.

